# Bioerodable PLGA-Based Microparticles for Producing Sustained-Release Drug Formulations and Strategies for Improving Drug Loading

**DOI:** 10.3389/fphar.2016.00185

**Published:** 2016-06-28

**Authors:** Felicity Y. Han, Kristofer J. Thurecht, Andrew K. Whittaker, Maree T. Smith

**Affiliations:** ^1^Centre for Integrated Preclinical Drug Development, The University of QueenslandBrisbane, QLD, Australia; ^2^Australian Institute for Bioengineering and Nanotechnology, The University of QueenslandBrisbane, QLD, Australia; ^3^Centre for Advanced Imaging, The University of QueenslandBrisbane, QLD, Australia; ^4^ARC Centre of Excellence in Convergent BioNano Science and TechnologyBrisbane, QLD, Australia; ^5^School of Pharmacy, The University of QueenslandBrisbane, QLD, Australia

**Keywords:** PLGA microparticles, drug delivery system, hydrophilic molecule, biodegradation mechanisms, tuneable release, microfluidics, supercritical carbon dioxide, hydrogel template

## Abstract

Poly(lactic-*co*-glycolic acid) (PLGA) is the most widely used biomaterial for microencapsulation and prolonged delivery of therapeutic drugs, proteins and antigens. PLGA has excellent biodegradability and biocompatibility and is generally recognized as safe by international regulatory agencies including the United States Food and Drug Administration and the European Medicines Agency. The physicochemical properties of PLGA may be varied systematically by changing the ratio of lactic acid to glycolic acid. This in turn alters the release rate of microencapsulated therapeutic molecules from PLGA microparticle formulations. The obstacles hindering more widespread use of PLGA for producing sustained-release formulations for clinical use include low drug loading, particularly of hydrophilic small molecules, high initial burst release and/or poor formulation stability. In this review, we address strategies aimed at overcoming these challenges. These include use of low-temperature double-emulsion methods to increase drug-loading by producing PLGA particles with a small volume for the inner water phase and a suitable pH of the external phase. Newer strategies for producing PLGA particles with high drug loading and the desired sustained-release profiles include fabrication of multi-layered microparticles, nanoparticles-in-microparticles, use of hydrogel templates, as well as coaxial electrospray, microfluidics, and supercritical carbon dioxide methods. Another recent strategy with promise for producing particles with well-controlled and reproducible sustained-release profiles involves complexation of PLGA with additives such as polyethylene glycol, poly(ortho esters), chitosan, alginate, caffeic acid, hyaluronic acid, and silicon dioxide.

## Introduction

Drug delivery systems with high efficiency and tuneable release characteristics continue to be sought. This is despite recent advances in the field of nanobiotechnology that have produced a range of new materials for improving control over drug delivery rates (Hillery et al., 2005). The strategies used to produce these sustained-release dosage forms involve drug loading of biodegradable polymeric microspheres and have the potential to provide a more facile route to adjust release rates (Kapoor et al., 2015).

Poly(lactic-co-glycolic acid) (PLGA), is a widely used biodegradable material use for encapsulation of a broad range of therapeutic agents including hydrophilic and hydrophobic small molecule drugs, DNA, proteins, and the like (Zheng, 2009; Malavia et al., 2015), due to its excellent biocompatibility (Barrow, 2004; Kapoor et al., 2015). Complete release of encapsulated molecules is achieved via degradation and erosion of the polymer matrix (Anderson and Shive, 1997, 2012; Fredenberg et al., 2011). Importantly, PLGA is generally recognized as safe by international regulatory agencies such as the United States Food and Drug Administration (FDA) and the European Medicines Agency (EMA) for use in pharmaceutical products administered to humans via conventional oral and parenteral routes (Yun-Seok et al., 2010) as well as suspension formulations for implantation without surgical procedures (Freiberg and Zhu, 2004).

However, factors limiting more widespread use of PLGA in pharmaceutical products include relatively low drug loading efficiency, difficulties in controlling encapsulated drug release rates and/or formulation instability (Varde and Pack, 2004; Freitas et al., 2005; Yun-Seok et al., 2010; Ansari et al., 2012; Danhier et al., 2012; Reinhold and Schwendeman, 2013). In the following sections, we review strategies and new technologies with promise for addressing these issues.

## Challenges in improving drug loading of microparticles with acceptable control over release rate profiles

### Physicochemical properties of the incorporated drug(s)

Achieving the desired loading of low molecular weight (*M*_*r*_), hydrophilic molecules in polymeric particles is more difficult than for hydrophobic small molecules, despite the large number of micro-encapsulation methods described in peer-reviewed publications and patents (Ito et al., 2011; Ansari et al., 2012). Manipulation of the physicochemical properties is often the most effective means for optimizing drug loading into PLGA microspheres (Curley et al., 1996; Govender et al., 1999). For example, small molecules that are hydrophilic in their salt form can be converted to the corresponding free acid or free base forms that are more hydrophobic, subsequently leading to higher drug loading (Han et al., 2015). The physicochemical properties of the incorporated drug(s) also significantly affect release rate profiles (Hillery et al., 2005).

For PLGA microparticles, release of the encapsulated drug occurs via diffusion and/or homogeneous bulk erosion of the biopolymer (Siegel et al., 2006; Kamaly et al., 2016) with the diffusion rate dependent upon drug diffusivity and partition coefficient (Hillery et al., 2005). These parameters are influenced by the physicochemical properties of the drug, such as molecular size, hydrophilicity, and charge (Hillery et al., 2005). A relatively high content of a water-soluble drug facilitates water penetration into particles and formation of a highly porous polymer network upon drug leaching (Feng et al., 2015). By contrast, hydrophobic drugs can hinder water diffusion into microparticulate systems and reduce the rate of polymer degradation (Klose et al., 2008). This is illustrated by observations that for six drugs with diverse chemical structures, viz. thiothixene, haloperidol, hydrochlorothiozide, corticosterone, ibuprofen and aspirin, there were significant between-molecule differences in release rate from PLGA (50:50) pellets, despite their similar drug loading at 20% by weight (Siegel et al., 2006). Hence, the design of biodegradable polymeric carriers with high drug loading must take into consideration the effects of the encapsulated drug itself on the mechanisms underpinning biopolymer degradation that influence release rate (Siegel et al., 2006).

### Particle size

Key factors in the design of microparticle drug delivery systems include microsphere size and morphology (Langer et al., 1986; Shah et al., 1992; Mahboubian et al., 2010) as these parameters potentially affect encapsulation efficiency (EE), product injectability, *in vivo* biodistribution, and encapsulated drug release rate (Nijsen et al., 2002; Barrow, 2004), efficacy and side-effect profiles (Liggins et al., 2004). Typically, optimal release profiles are achieved by using microspheres with diameters in the range, 10–200 μm (Anderson and Shive, 1997). For particle diameters < 10 μm, there is a risk that microspheres will be phagocytosed by immune cells (Dawes et al., 2009). On the other hand, microspheres >200 μm may cause an immune response and inflammation (Dawes et al., 2009).

For large diameter particles, the small surface area per unit volume leads to a reduced rate of water permeation and matrix degradation relative to smaller particles and so the maximum possible rate of encapsulated drug release is reduced (Dawes et al., 2009). For drugs microencapsulated in larger microparticles, duration of action is potentially longer due to higher total drug loading and a longer particle degradation time (Klose et al., 2006). Hence, a good understanding of the relationship between biopolymer composition, microparticle morphology and size is essential for tailored production of particulate materials with pre-determined drug release profiles (Cai et al., 2009). However, based upon the diversity of encapsulated drug release profiles produced by PLGA microspheres of varying sizes to date (Table 1), release rates do not necessarily conform to predicted behavior and it is only possible to quantitatively predict the effect of microparticle size on drug release kinetics for certain well-defined formulations (Siepmann et al., 2004).

Table 1**Influence of particle size, polymer physicochemical properties as well as PLGA composition on drug loading and release profiles**.

**Table:** 

**(1) Particle size**				
Drug loading and release rates from PLGA particles do not necessarily conform to predicted behavior as the effect of microparticle size on drug release kinetics quantitatively can only be predicted for certain well-defined formulations.				
**Encapsulated drug**	**Particle size (μm)**	**Drug loading or EE**	**Drug release profile**	**References**
Lidocaine	Increase from 20 to 50 to 120	N/A	Release rate ↓ as particle size ↑	Klose et al., 2006
Huperzine A	Increase from 125–200 to 200–400 to 400–700	EE ↑	Release rate ↓ as particle size ↑	Fu et al., 2005
Dexamethasone	1.0	11%	Slow-release particles but with initial burst release	Dawes et al., 2009
	20	1%	Sustained release over a 550 h period	
5-fluorouracil	70–120	35%	~90% release in 7 days	Siepmann et al., 2004
	20	20%	90% release over 21days	
Drug-free	< 50, < 20 and < 1 (each size prepared by a different process)	N/A	At pH 7.4 and 37°C, ↑ polymer degradation rate for larger microspheres	Dunne et al., 2000

**Table:** 

**(2) Physicochemical properties of the biopolymer**				
The hydrophilicity or hydrophobicity of PLGA end-groups affect hydration during the pore diffusion phase thereby influencing the rate of drug release from the polymeric matrix. PLGA composition-dependent changes to microparticle morphology may also affect encapsulated drug release profiles.				
**Encapsulated drug**	**PLGA Composition**	**Effect on particle size, drug loading and release profile**	**References**
FITC-dextran	PLGA (50:50) with a carboxylic acid-end group, viz RG503H (*M*r** 24000-38000)	Sustained release achieved by ↑ porosity, pore size, and loading	Cai et al., 2009
	PLGA (50:50) with an ester-end group, viz RG502 (*Mr* 7000–17000)	Porosity and pore size had a minimal effect on release profile beyond initial release	
Huperzine A	PLGA (75:25) of varying *M*r**, viz 15, 20, and 30 kDa	Drug loadings of 3.53, 1.03, and 0.41% respectively; inversely correlated with *M*r**	Fu et al., 2005; Ansari et al., 2012
Cephalexin	↑ Concentration of PLGA in the organic solvent (chloroform) from 25 to 33.3 mg/ml	Higher drug loading and larger particle size	Wasana et al., 2009

**Table:** 

**(3) Recent advances with promise for improving PLGA delivery systems**					
**Methods**	**Encapsulated drug**	**Particle size (μm)**	**Drug loading or EE**	**Drug release profile**	**References**
Hydrogel template	OHR1031	60 ± 10	57% w/w, ~100% EE	Nearly zero-order for over 3 months, with no initial burst, which was desirable	Malavia et al., 2015
	Felodipine, Paclitaxel, Progesterone and Risperidone	10–50	50–65%	Sustained release profiles	Acharya et al., 2010b
scCO_2_ in combination with a w/o/o/o method	Dexamethasone phosphate	70–80	90% EE	Sustained release profile without initial burst release	Thote and Gupta, 2005
scCO_2_	hGH	~61		Controlled release for > 7 days	Jordan et al., 2010
	Tetanus toxoid (TT)			Single injection TT-loaded PLA particles in mice antibody titres similar to those evoked by multiple injections of a commercial alum-adsorbed TT vaccine was produced	Baxendale et al., 2011
Coaxial electrospray (CES)	Levetiracetam	Double-layered: release over 18-days whereas encapsulation in classical core-shell fibers gave linear release for 4 days followed by steady-state			Viry et al., 2012
	Growth factors	Controlled-release: Coaxial electrospinning of biodegradable core-shell structured microfibrous scaffolds using PLGA as the shell and hyaluronic acid as the core			Joung et al., 2011
	Multiple drugs	Coaxial tri-capillary electrospray system produced monodispersed PLGA-coated particles containing multiple drugs in one step			Lee et al., 2011
Spray drying	Double-layered enzyme-triggered release in the gastrointestinal tract: Negligible loss of the core in the gastric environment with gradual release of the core in the intestinal environment without initial burst release				Park et al., 2014
Polymer self-healing	Spontaneous pore closure (or self-healing) of PLGA microparticles at temperatures greater than the polymer glass transition temperature is used to microencapsulate biomacromolecules (proteins, peptides, and polysaccharides) in aqueous media. This approach avoids exposure to organic solvents that would otherwise occur during PLGA conventional encapsulation and uses mild processing conditions, that together minimize damage to encapsulated naked DNA, proteins, etc.				Reinhold and Schwendeman, 2013

**Table:** 

**(4) Various additives complexing with PLGA with increased drug loading and/or sustained release profiles**				
**Additives**	**Encapsulated drug**	**Drug loading or EE**	**Drug release profile**	**References**
POE/PLGA	BSA	9–11% and EE 60–90%	95% over 30 days	Shi et al., 2003
POE/PLGA	Cyclosporin A	6–10% and EE 60–90%	14% over 15 days followed by 78% over the next 27 days	Shi et al., 2003
Alginate and chitosan-PLGA double walled	BSA	EE at 75% *c.f.* 65% compared with single-walled systems	5–10% in 30 min *c.f.* 30% for single-walled systems	Zheng and Liang, 2010
Alginate-PLGA double walled	Metoclopramide HCl	EE increase from 30% to 60% *c.f.* single walled system	Improved release profile	Lim et al., 2013
4% w/w chitosan/PLGA	Resveratrol	EE 40–52% Particle size: 11 to 20 μm and more stable	Improved controlled release	Sanna et al., 2015
Caffeic acid grafted PLGA (g-CA-PLGA)	Ovalbumin	EE increased from 35 to 95% *c.f.* PLGA alone (size 15–50 μm)	Unchanged	Selmin et al., 2015
Mixed copolymer of PLGA 50:50 (*M*r** 100,000 and 14,000) 1:7	Pentamidine	23.7%, whereas only 9.8 and 13.9 %, when prepared with either of them alone	Produced microcapsules with desired release profiles	Graves et al., 2004
Aqueous core-PLGA shell	Risedronate sodium	2.5-fold increase: 31.6% *c.f.* 12.7% for classical PLGA microspheres	Sustained release according to diffusion-controlled Higuchi model	Abulateefeh and Alkilany, 2015
Porous silicon oxide (pSiO_2_)-PLGA	Daunorubicin	Slightly increased loading (3.1–4.6%) *c.f.* 2.7% for PLGA-daunorubicin microspheres	A 2-5 fold longer duration of release *c.f.* PLGA-daunorubicin microspheres	Nan et al., 2014

BSA, Bovine serum albumin; EE, Encapsulation efficiency; hGH, Human growth hormone; M_r_, Molecular weight; OHR1031, a small molecule for the treatment of glaucoma; PLA, poly(lactic acid); PLGA, poly(lactic-co-glycolic acid); POE, poly(ortho esters).

### Biodegradation mechanisms of PLGA-microparticles

The two main mechanisms that drive drug release from PLGA microspheres are diffusion and degradation/erosion (Kamaly et al., 2016). For PLGA (50:50) particles, drug release occurs in two phases. In the first phase, there is a rapid decrease in molecular weight (*M*_*r*_) but little mass loss whereas in the second phase, the opposite occurs. This indicates that PLGA particle degradation involves heterogeneous mechanisms and that drug release is underpinned primarily by diffusion rather than polymer degradation (Engineer et al., 2010).

PLGA is a typical bulk-eroding biopolymer such that water permeates readily into the polymer matrix forming pores so that degradation takes place throughout the microspheres (Varde and Pack, 2004). Comparison of encapsulated drug release profiles from surface eroding biopolymers such as poly(ortho esters) (POE) and polyanhydrides with bulk-eroding biopolymers such as PLGA, is lacking. Hence, future research addressing this knowledge gap is needed to better inform design of microparticle formulations with the desired release profiles (Engineer et al., 2010) that may potentially include formulations comprising mixed bulk and surface-eroding biopolymers (Feng et al., 2015).

### Physicochemical properties of the biopolymer

For drugs encapsulated in PLGA microparticles, the desired release rates can be achieved by adjusting the ratio of lactic acid to glycolic acid and by altering the physicochemical properties [e.g., *M*_*r*_, end-group (ester or carboxylic) functionality] that influence microparticle morphology (Table 1; Mao et al., 2007; Cai et al., 2009; Gasparini et al., 2010; Nafissi-Varcheh et al., 2011). The physical properties of PLGA particles are also dependent upon the drug delivery device size, exposure to water (surface shape), as well as storage temperature and humidity (Table 1) (Houchin and Topp, 2009). These properties not only affect the ability of the biopolymer to be formulated but also influence its degradation rate (Table 1; Makadia and Siegel, 2011). Another factor that contributes to encapsulated drug release from PLGA microspheres is the concentration of polymer in the organic solvent during formulation (Wasana et al., 2009).

### Choice of surfactant

During microparticle formulation using conventional solvent evaporation methods, an emulsifier is required to ensure droplet stability until the polymer concentration in the organic solvent is sufficiently high to maintain particle conformation (Chemmunique, 1980; Hwisa et al., 2013). The most widely used emulsifier in the preparation of PLGA micro/nanoparticles is poly (vinyl alcohol) (PVA) (Wang et al., 2015). It is worth noting that D-α-tocopheryl polyethylene glycol 1000 succinate (vitamin E TPGS; FDA-approved as a water-soluble vitamin E nutritional supplement) markedly improved drug loading at a concentration an order of magnitude lower (0.3 mg/ml) than analogous systems that used PVA (5 mg/ml) (Feng et al., 2007).

### Methods for producing microparticles for sustained-release formulations

Drugs, including many small molecules, that are soluble in the polymer solution, can be encapsulated by simply co-dissolving with the polymer for the most commonly used methods (Table 2).

**Table 2:** **Methods for producing PLGA based microparticles for sustained-release formulations: Advantages and Disadvantages**.

**Methods**	**Schematic diagrams**	**• Advantages**	**• Disadvantages**	**References**
Oil-in-water (o/w) emulsion	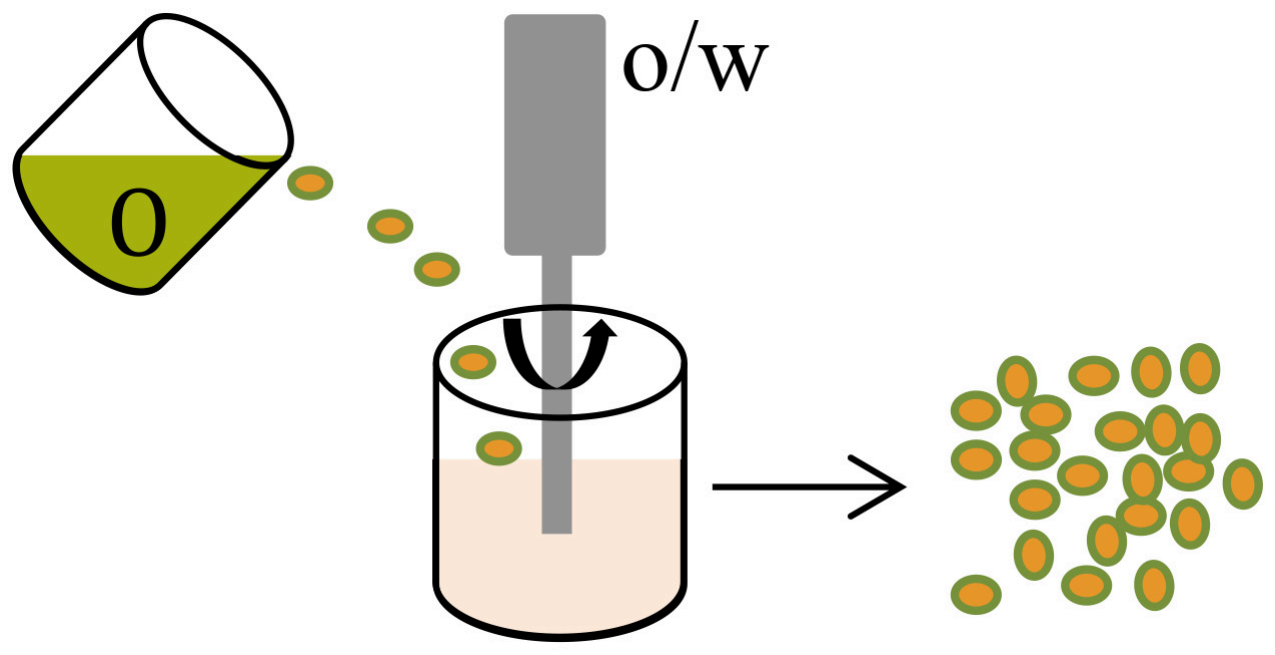	Simplicity Suitability for temperature-sensitive compounds Control of particle size	Low encapsulation efficiency especially for water-soluble payloads Solvent residuals Low yield, agglomeration of sticky particles	Varde and Pack, 2004
Water-in-oil-in-water (w/o/w) emulsion	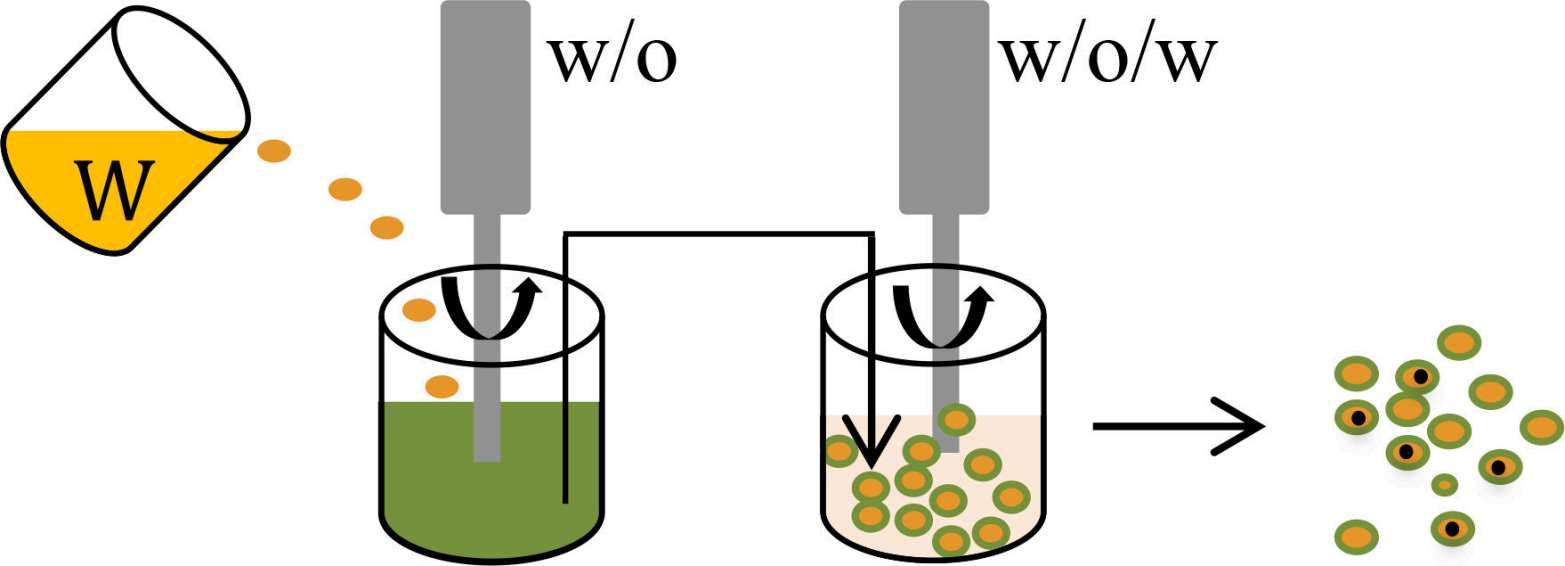			
Supercritical CO_2_ (scCO_2_)	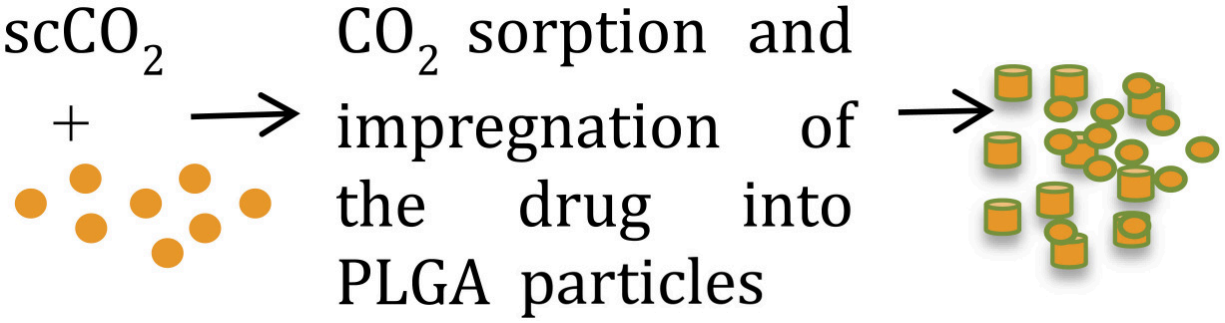	Negligible residual organic solvent	Multiple steps, poor control of particle size, size distribution, and morphology	Falco et al., 2012; Dhanda et al., 2013
Spray drying	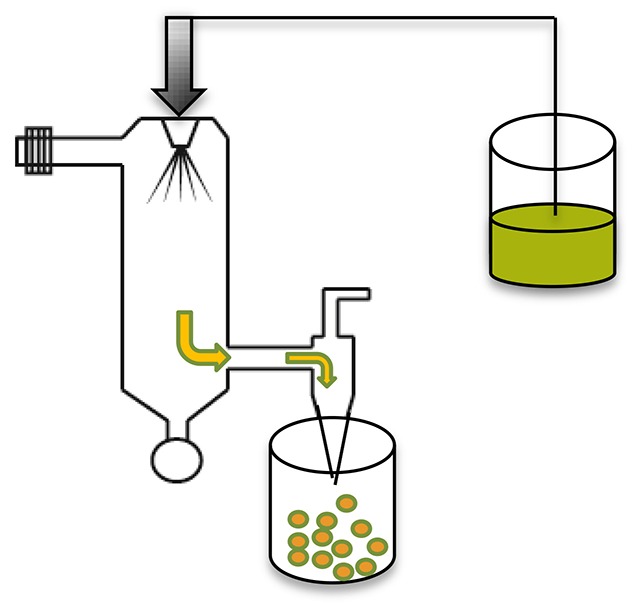	Can encapsulate wide range of drugs/peptides/proteins into microparticles without significant loss Final drying step not required One step and reproducible Atomizers (nozzles) eliminate the need for complicated pre-preparation processes and enable continuous manufacture by utilization of liquid feeds *via* two separate channels	Adhesion of microparticles to inner walls of the spray-dryer Not suitable for temperature-sensitive compounds Difficult to control particle size Low yield, agglomeration of sticky particles	Makadia and Siegel, 2011; Sosnik and Seremeta, 2015; Wan and Yang, 2016
CES (Other modification, such as, coaxial tri-capillary electrospray, Emulsion-coaxial electrospinning)	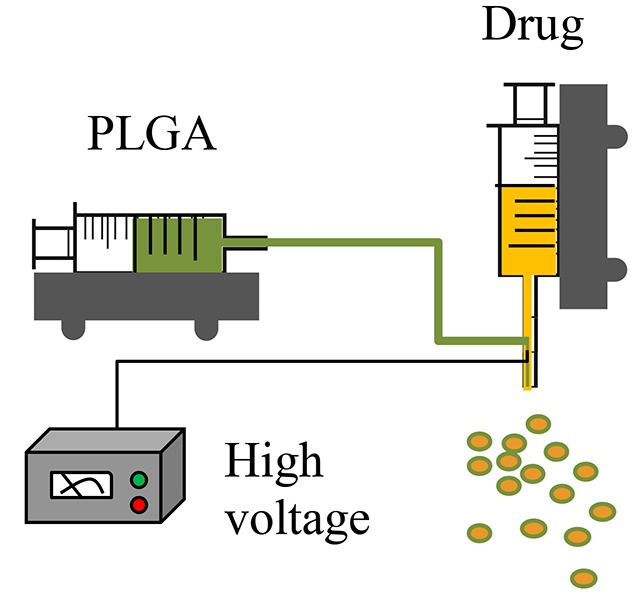	Nearly 100% encapsulation rate Useful for encapsulating water-soluble molecules Protects biologically active payloads from processing-induced damage Potential to control particle morphology with flexibility and reproducibility for both micro- and nanoparticle size ranges	At early stage; requires further development Standardized protocols and systematic process controls not available as yet Lack of an effective particle collection method; commonly used one-step collection methods cannot facilitate shell hardening, or maintain particle morphology or prevent particle aggregation Lack of a more productive nozzle design	Lee et al., 2011; Viry et al., 2012; Zhang et al., 2012; Zamani et al., 2014; Yuan et al., 2015
Microfluidics (Other modification, such as, capillary microfluidics coupled with solvent evaporation)	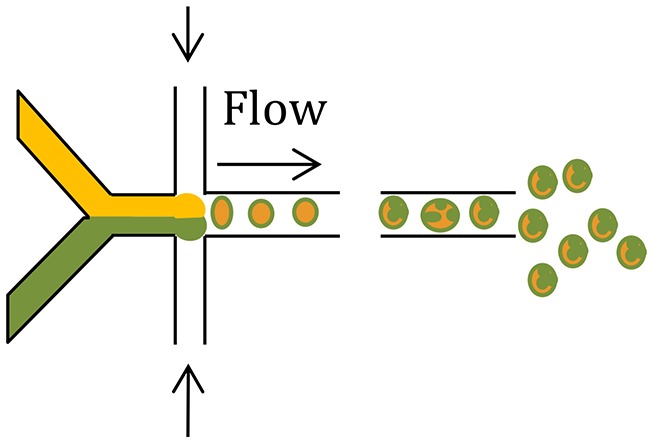	Ultra-small quantities of reagents needed Precise control over drug release rate, drug loading efficiency, particle shell thickness, particle shape and size Multiple components are easily generated using single-step emulsification	A time-consuming method as single drops are generated one at a time	Demello, 2006; Hung et al., 2010; Xie et al., 2012; Cho and Yoo, 2015; Leon et al., 2015
Hydrogel template	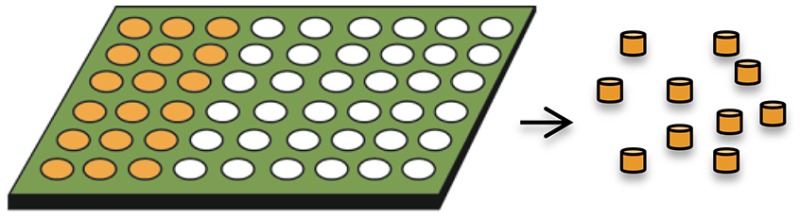	Higher drug loading and sustained release profiles	novel technique not widely used as yet	Acharya et al., 2010a,b; Malavia et al., 2015

CES, Coaxial electrospray; PLGA, Poly(lactic-co-glycolic acid).

For the water-soluble salts of small molecule drugs, encapsulation efficiency can be improved by their conversion to a hydrophobic form, such as by complexation with ionic surfactants (Cohen et al., 1991) or to the corresponding free acid or free base form (Han et al., 2015). Alternative approaches include suspension of solid (e.g., lyophilized) particulates in the polymer solution; or use of a water-in-oil-in-water (w/o/w) solvent evaporation (double-emulsion) method. When using a w/o/w method, relatively higher drug loading and reproducible sustain-release profiles can be achieved by formulations that have a smaller volume for the inner water phase (Wasana et al., 2009; Chaudhari et al., 2010), a low preparation temperature (Yang et al., 2000; Fu et al., 2005; Chaudhari et al., 2010; Ito et al., 2011) and a suitable pH of the external phase (Bodmeier and Mcginity, 1988; Govender et al., 1999; Leo et al., 2004).

Newer technologies and approaches for achieving high levels of drug loading with suitable sustained release profiles are reviewed in the following sections and compared in Tables 1, 2.

## Recent advances with promise for improving PLGA-based drug delivery systems

### Hydrogel templates

Hydrogel templates enable high drug loading (~50%) and high incorporation efficiencies (~100%) to be achieved and are amenable to small molecules and biologics (Tables 1, 2) (Malavia et al., 2015). Any water insoluble material can be used as the microparticle matrix to produce the desired drug release profiles, and microparticles are recovered from the readily soluble hydrogel templates. The technology allows for precise control of the size and shape of template wells in every dimension so that microparticles with a narrow size distribution can be produced (Lu et al., 2014; Malavia et al., 2015). These attributes enable sustained-release microparticles to be produced for injection using narrow bore needles into sensitive spaces such as the eye, with nearly zero-order drug release for over 3 months with virtually no initial burst release (Malavia et al., 2015). However, more research is needed to better understand the effect of microparticle size and shape on encapsulated drug release kinetics and *in vivo* performance for a broad range of molecules with widely differing physicochemical properties.

### Coaxial electrospray

Coaxial electrospray (CES) produces double-layered microparticles using an electric field applied to both the outer (PLGA carrier) and the inner (drug loaded) solutions sprayed simultaneously through two separate feeding channels of a coaxial needle into the one nozzle (Yuan et al., 2015). At a certain voltage threshold, a conical shape (e.g., “Taylor cone”) forms and the jets of liquids (both inner and outer flows) are broken into double-layered microparticles (Yuan et al., 2015). In the CES process, a compound Taylor cone with a core-shell structure is formed on top of the spray nozzle, and the outer polymeric solution encapsulates the inner liquid (Yuan et al., 2015). The bulk liquid is broken into small charged droplets by coulombic repulsion (Yuan et al., 2015). Using this technique, parameters such as orientation of the jets, material flow rates, and rate of solvent extraction can be controlled to create uniform and well-centered double-walled microspheres exhibiting a controllable shell thickness (Makadia and Siegel, 2011). The CES process enables effective encapsulation of proteins, drugs, and contrast agents with high efficiency, minimal loss of biological viability, and excellent control of core-shell architecture (Tables 1, 2) (Zamani et al., 2014; Yuan et al., 2015).

### Microfluidic fabrication

Microfluidic devices use electrostatic forces to control the size and shape of particles for enhanced tuning of drug release characteristics (Zhang et al., 2013). Microfluidic systems have been employed for fabrication of complex drug carriers with precise size and composition leading to a predictable and tuneable release profile (Tables 1, 2) (Leon et al., 2015; Riahi et al., 2015). Two continuous and immiscible streams (i.e., oil and water) are infused via two separate inlets (Xu et al., 2009). Monodisperse droplets are generated at the junction where the two streams meet due to the high shear stress. The droplet sizes are in the range 20–100 μm (Xu et al., 2009) and 100–300 nm (Xie et al., 2012). In contrast to the classical double emulsion methods, multiple components are easily generated by a single-step emulsification in the microfluidic device (Xie et al., 2012). By introducing the second stream, droplets may be re-encapsulated which is useful for preparing core-shell structures (Nie et al., 2006).

A novel and versatile microfluidic approach for fabrication of PLGA/PCL Janus and microcapsule particles involves changing the organic solvent of the dispersed phase from dimethyl carbonate to dichloromethane (Li et al., 2015). The shell on the microcapsule particle surface is comprised of PLGA only, and the core is comprised of PCL in which tiny PLGA beads are embedded (Li et al., 2015). Interestingly, the Janus and microcapsule particles exhibited distinct degradation behaviors, implying their potential for differential effects on drug delivery and release profiles (Li et al., 2015).

### Supercritical CO_2_

Supercritical CO_2_ (scCO_2_) provides a “green” alternative to traditional microparticle formulation techniques as it avoids use of toxic organic solvents or elevated temperatures (Tables 1, 2) (Budisa and Schulze-Makuch, 2014). Owing to the very short encapsulation process (5–10 min) at a relatively low temperature and modest pressure, and absence of organic solvents, the activity of bioactive molecules including proteins is maintained (Howdle et al., 2001; Koushik and Kompella, 2004; Della Porta et al., 2013). Because the complete process is anhydrous, it can be used to produce sustained-release formulations of multiple hydrophilic molecules (Thote and Gupta, 2005).

New variations to the use of scCO_2_ technology take advantage of other properties of CO_2_ such as its capacity to extract active pharmaceutical ingredients (APIs) from natural compounds or to form polymers (Champeau et al., 2015). New protocols under development hold promise for fabricating drug-eluting implants using a scCO_2_ impregnation process (Champeau et al., 2015).

### Spray drying

Drug/protein/peptide loaded microspheres can be prepared by spraying a solid-in-oil dispersion or water-in-oil emulsion in a stream of heated air without significant losses (Makadia and Siegel, 2011). The type of drug (hydrophobic or hydrophilic) for encapsulation informs the choice and nature of the solvent to be used, whereas the temperature of the solvent evaporation step and feed rate affect microsphere morphology (Tables 1, 2) (Makadia and Siegel, 2011). Various spray drying techniques have been reported and are reviewed elsewhere (Wan and Yang, 2016).

### Polymer self-healing

“Self-healing” is a phenomenon whereby polymers with damaged structures (e.g., pores, cracks, and dents), undergo spontaneous rearrangement of the polymer chains to produce healing (repair) (Syrett et al., 2010). This is important because pore closure in PLGA microparticles at physiological temperature impedes the pore-diffusion pathway and greatly reduces initial burst release of a micro-encapsulated peptide (Wang et al., 2002). Similarly, porous PLGA microspheres loaded with recombinant human growth hormone (rhGH) prepared by the solvent evaporation technique and using the surfactant pluronic F127 as porogen, underwent pore closure at the polymer surface following solvent exposure (Kim et al., 2006). These “healed” non-porous microspheres exhibited sustained drug release profiles over an extended period (Kim et al., 2006). The post-healing approach can be used to overcome shear-induced microparticle degradation, solvent-associated erosion of delicate core materials, or unexpected payload release during emulsification (Tables 1, 2) (Na et al., 2012). Strategies for “healing” pores in the microparticle surface include solvent swelling, or infrared irradiation which is potentially an even milder approach for inducing self-healing (Na et al., 2012).

### Complexing PLGA with additives

As noted in an earlier section of this review, the chemical composition of PLGA-particulate drug delivery systems greatly influences their physicochemical properties, and this in turn governs the biodistribution and pharmacokinetics of the encapsulated drug (Zhang et al., 2013). Hence, complexation of PLGA with suitable additives (Table 1) including poly(ethylene glycol) (PEG), POE, chitosan and/or alginate, caffeic acid, hyaluronic acid, TPGS, and SiO_2_ (Shi et al., 2003; Graves et al., 2004; Zheng and Liang, 2010; Lim et al., 2013; Navaei et al., 2014; Abulateefeh and Alkilany, 2015; Sanna et al., 2015; Selmin et al., 2015; Wang et al., 2015), may lead to higher drug loading and the desired sustained release profile (Shi et al., 2003; Graves et al., 2004; Zheng and Liang, 2010; Lim et al., 2013; Navaei et al., 2014; Abulateefeh and Alkilany, 2015; Sanna et al., 2015; Selmin et al., 2015).

Other strategies with promise for improving controlled-release drug delivery systems include double walled/layered PLGA (Navaei et al., 2014) and nanoparticles-in-microparticles (Lee et al., 2013). Additionally, polymer-brush PLGA-based drug delivery systems appear promising due to the versatility and controllability of the method for controlling particle shape (Huang et al., 2014).

## Conclusions

In the past decade, considerable progress has been made on addressing the issues of (i) low drug loading, (ii) particle instability, and (iii) adequate control of drug release profiles for PLGA-based microparticle drug delivery systems. Strategies for increasing drug loading in PLGA-microspheres include modification of the classical solvent evaporation methods, preparation of multi-layered microparticles, and development of novel methods for microparticle fabrication including hydrogel templates, coaxial electrospray, microfluidics, and scCO_2_. Additionally, methods involving complexation of PLGA with additives such as PEG, POE, chitosan and/or alginate, caffeic acid, hyaluronic acid and SiO_2_, appear promising. Nevertheless, there is a great need for innovation in development of time-efficient methods for controlling the factors that influence drug loading and release profiles as a means to inform the design of next-generation controlled-release drug delivery systems (Draheim et al., 2015).

## Author contributions

All authors listed, have made substantial, direct and intellectual contribution to the work, and approved it for publication.

## Funding

FH is supported by a postdoctoral fellowship funded by a National Health and Medical Research Council (NHMRC) grant, APP1107723.

### Conflict of interest statement

The authors declare that the research was conducted in the absence of any commercial or financial relationships that could be construed as a potential conflict of interest.
